# Self-Reported Chronic Back Pain and Current Depression in Brazil: A National Level Study

**DOI:** 10.3390/ijerph20085501

**Published:** 2023-04-13

**Authors:** Ryan J. Norris, S. Cristina Oancea, Luciana B. Nucci

**Affiliations:** 1 Department of Pathology, School of Medicine and Health Sciences, University of North Dakota, Grand Forks, ND 58202, USA; 2 Department of Anesthesiology, School of Medicine, Wake Forest University, Winston-Salem, NC 27109, USA; 3 Department of Population Health, School of Medicine and Health Sciences, University of North Dakota, Grand Forks, ND 58202, USA; 4 Health Sciences Post Graduate Program, School of Life Sciences, Pontifical Catholic University of Campinas (PUC-Campinas), Campinas 130869-00, SP, Brazil

**Keywords:** depression, chronic back pain, PNS 2019, national health survey, Brazil

## Abstract

There is limited literature investigating the association between chronic back pain (CBP) and depression in Brazil. This study evaluates the association between CBP, CBP-related physical limitations (CBP-RPL), and self-reported current depression (SRCD), in a nationally representative sample of Brazilian adults. The data for this cross-sectional study came from the 2019 Brazilian National Health Survey (*n* = 71,535). The Personal Health Questionnaire depression scale (PHQ-8) was used to measure the SRCD outcome. The exposures of interest were self-reported CBP and CBP-RPL (none, slight, moderate, and high limitation). Multivariable weighted and adjusted logistic regression models were used to investigate these associations. The weighted prevalence of SRCD among CBP was 39.5%. There was a significant weighted and adjusted association between CBP and SRCD (weighted and adjusted odds ratio (WAOR) 2.69 (95% CI: 2.45–2.94). The WAOR of SRCD among individuals with high, moderate, and slight levels of physical limitation was significantly greater than for those without physical limitation due to CBP. Among Brazilian adults with high levels of CBP-RPL, there was over a five-fold increased risk of SRCD compared to those without CBP-RPL. These results are important for increasing awareness of the link between CBP and SRCD and for informing health services policies.

## 1. Introduction

Depression is the number one cause of disability in the world, with approximately 280 million people suffering worldwide [1]. The current (5th) edition of the Diagnostic and Statistical Manual of Mental Disorders (DSM-5) defines major depression as specific episodes of at least 2 weeks duration that involve clearly distinguishable changes in cognition, affect, and neurovegetative functions, with remission commonly being seen between episodes [2]. Prevalence estimates of depression are based on validated questionnaires that individuals self-administer or are clinician-administered [3]. In 2014, it was estimated that over 15.5 million United States (US) adults ages 18 or older (6.6% of the US population) had suffered at least one major depressive episode in the prior year [4]. In an international epidemiological study performed in 1996 using the DSM-IV’s definition of major depressive episodes in 18 countries, over 53% of participants in ten high-income countries and 54% of participants in eight low- to middle-income countries screened positive for a major depressive episode. Specifically, Brazil reported the second-highest prevalence of all 18 countries at 66.0% [5]. Depression wreaks sufficient havoc on one’s life on its own but when it occurs as a comorbidity, a significant increase in disability, morbidity, and mortality occurs [6]. The relationship between depression and several comorbid conditions appears to be bidirectional—depression has been shown to predict the development of future chronic diseases along with worse medical outcomes when problems such as heart disease arise, while at the same time, serious physical illness is associated with a higher prevalence of depression [6,7,8,9,10,11,12,13,14].

In 2013, the Global Burden of Disease study collaborators found that back pain was the number one cause of the global burden of disease as measured by years lived with disability, with major depression coming in second [15]. Chronic pain is defined as pain that persists past normal healing time, typically lasting or recurring for more than three to six months [16]. Common causes of back pain include muscle strain, disc herniation, lumbar spondylosis, spinal stenosis with neurogenic claudication, spondylolisthesis, spondylolysis, ankylosing spondylitis, infection, malignancy, cauda equina syndrome, conus medullaris syndrome, vertebral compression fracture, and trauma [17]. Across the world, low back pain has an annual prevalence of 38% and a lifetime prevalence of 39% [18]. In Brazil, almost 1 in 5 adults reported chronic back pain (CBP) in 2013, the second most frequent chronic condition reported after hypertension [19].

The link between CBP and depression has been extensively researched around the world. In a large cross-sectional study looking at depression with psychotic experiences and its association with chronic physical conditions across 47 low- and middle-income countries on the 2002–2004 World Health Survey, depression was found to be significantly associated with CBP (*p* < 0.0001) [7]. In another multi-national study, Vancampfort et al., reported a significant direct and total effect of chronic back pain and depression among middle- and older-aged adults in six low- and middle-income countries [20]. Rush et al., conducted a literature review in 2000 and found that CBP appears to be associated with major depression in around 50% of cases [11]. While Adilay et al., reported a non-significant association between depression and back pain in their study of 75 patients in Turkey, other studies conducted in Spain, Pakistan, Australia, Japan, and the Netherlands have found significant associations between the two [8,9,13,21,22,23].

Clearly, the link between depression and CBP has been well-documented throughout the world. However, it has only been studied previously in Brazil on a very limited scale and with contradicting results. Trocoli and Botelho found no association between CBP and depression among 65 patients who presented to a walk-in clinic with back pain in São Paulo, Brazil in 2015 [24], while Rodrigues de Souza et al., found a significant association between depression and CBP in 2016 among 30 patients with back pain in São Carlos, Brazil [25].

Therefore, the current study has two main goals. First, to evaluate the association between self-reported CBP and self-reported current depression (SRCD) in a large, nationally representative sample of Brazilian adults. Second, to evaluate the association between the physical limitations due to CBP (none, slight, moderate, and high limitation) and SRCD. Both associations will be investigated while accounting for sociodemographic factors and depression comorbidities.

## 2. Materials and Methods

### 2.1. Study Sample

The sample for this study came from the “Pesquisa Nacional de Saúde” (PNS), a Brazilian national health survey completed in 2019 [26]. The PNS is a national household survey conducted by the Brazilian Ministry of Health in conjunction with the Brazilian Institute of Geography and Statistics (IBGE) [26]. The purpose of the study is threefold: (a) gather data on the health status and lifestyle of the population, (b) identify shortcomings with regards to access to health care, and (c) guide preventative interventions for various diseases, both chronic and infectious. The PNS sample was constructed via cluster sampling in three selection stages. In the first stage, the stratification of Primary Sampling Units (PSU) was conducted, consisting of census tracts or whole sectors, in which the selection was based on random home stratum. In the second stage, 10–14 households were randomly selected for each PSU. In the third stage, each household had a resident adult (18 years of age or older) randomly selected to be part of the PNS sample. The census tracts were identified and randomly selected based on the Integrated System of Household Survey—”Sistema Integrado de Pesquisas Domiciliares” (SIPD) from the IBGE and used as a “master sample’’ to reach most geographical locations in Brazil and obtain precise estimations [26,27].

**Figure 1:**
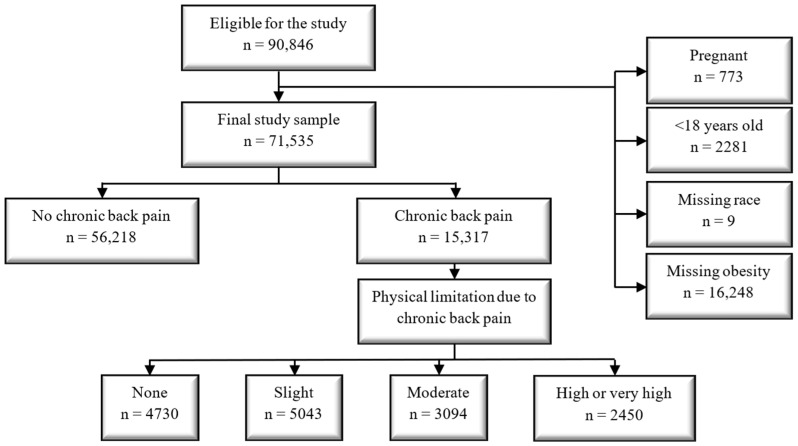
Final study sample cohort diagram.

Of the 90,846 individual interviews included in the PNS, pregnant women at the time of the survey (*n* = 773), individuals less than 18 years old (*n* = 2281), and those with missing variable values for race (*n* = 9) and for obesity (*n* = 16,248) were excluded. Pregnant women were excluded from the current analyses as depression is a known psychiatric disorder that may occur due to the pregnancy state [28,29,30], which is not the topic of the current study. Therefore, the final sample size used for the current study was 71,535 (Figure 1).

### 2.2. Outcome of Interest: Self-Reported Current Depression (SRCD)

Depression is often measured using the Patient Health Questionnaire depression scale (PHQ-9), a self-report version of the Primary Care Evaluation of Mental Disorders (PRIME-MD) that allows physicians to quickly screen patients for depression and follow-up on outcomes, given the severity score for each item [31]. The PHQ-9 has previously been validated for use in Brazil [32]. The PHQ-8 is an abbreviated version of the PHQ-9 that omits the question regarding thoughts of death or self-harm and has been shown to be a comparable measure of depression in both clinical and research settings, with a cut point of ≥10 being used to define SRCD [33,34,35,36].

### 2.3. First Exposure of Interest: Self-Reported Chronic Back Pain (CBP)

Self-reported CBP was assessed through answers to questions regarding the experience of chronic spine problems. No formal diagnosis was required or verified as the participant was merely asked if they had any chronic spine problems, such as chronic back or neck pain, lower back pain, sciatic pain, or problems with their vertebrae or intervertebral discs [37].

### 2.4. Second Exposure of Interest: Physical Limitation due to CBP (Slight, Moderate, and High Limitation)

Individuals who reported CBP were further questioned about the extent to which CBP limits their daily activities, such as working, performing household chores, etc. Potential answers were “no limitation”, “little limitation”, “moderate limitation”, “high limitation”, and “very high limitation” [37]. For the purposes of this study, “high limitation” includes responses of high and very high limitations of daily activities.

### 2.5. Covariates of Interest

There are a variety of demographic, social, and health risk factors associated with depression in adults, such as: being female [5,38,39,40], older age [38,41], unemployed [39,42], low educational attainment [39,41], lack of health insurance coverage [39,42], lack of participation in physical activity for leisure [43,44,45], and experiencing other chronic diseases [38,39,46]. These are also factors shown to be associated with CBP [47,48,49,50,51,52,53,54,55,56,57] and therefore are confounders of interest for the association under investigation.

The socio-demographic variables included in the current study are age, gender, race, education, and health insurance. Consideration of stable employment and income was not included in the current study, as the structure of the PNS rendered it difficult to ascertain. The primary comorbidities associated with both depression and CBP included herein are diabetes [52], hypertension [48,50], and obesity [47,48,50,51]. Obesity was calculated from self-reported weight and height and then calculated by dividing weight (kg) by height squared (m^2^). The cutoff point for obesity used in this study was a score of ≥30, in accordance with the National Institutes of Health’s clinical guidelines regarding obesity in adults [58].

### 2.6. Statistical Analysis

The Rao-Scott Chi-square test was used to look at basic comparisons between independent categorical variables and the outcome of interest, as that is the design-adjusted equivalent of Pearson’s Chi-square test. Weighted prevalence and 95% confidence intervals were calculated for the categorical variables. Multivariable weighted and adjusted logistic regression models were used to investigate the association between CBP and SRCD, as well as between physical limitations due to CBP and SRCD. All models were conducted in weighted unadjusted and adjusted format. Adjustment was made for age, gender, race, education, insurance, obesity, diabetes, and hypertension. SAS University Edition and SAS v4.0 (SAS Institute, Cary, NC, USA) were used to perform the statistical analyses. SAS survey procedures (proc surveyfreq, proc surveymeans, proc surveylogistic) were used to account for the complex sampling design. The level of statistical significance was set at 0.05.

## 3. Results

Overall, the final study participants had a median age of 42.3 years, with an interquartile range (IQR) of 30.5–55.9 years. The final sample size was 71,535 Brazilian adults, of which 51.2% were women. The majority of participants self-identified their race/ethnicity as white (46.0%). Only 30.2% of the participants had private health insurance and 44.5% had less than a high school education. In looking at the participants overall, the weighted prevalence of current depression was 10.3% (95% CI: 9.9–10.8%). Looking further by gender, the overall weighted percentage of SRCD was significantly higher in women (72.0%; 95% CI: 70.1–73.9%) than in men (28.0%; 95% CI: 26.1–29.9%). Among those participants with SRCD, 49.3% (95% CI: 47.2–51.3%) had completed less than a high school education, 73.7% (95% CI: 71.6–75.7%) had no private health insurance coverage, 8.7% (95% CI: 7.5–9.9%) reported being diagnosed with diabetes, 22.9% (95% CI: 21.2–24.6%) with hypertension, 78.0% (95% CI: 76.2–79.9%) did not participate in physical activity for leisure, and 28.0% (95% CI: 25.9–30.0%) were identified as being obese (Table 1). All of these prevalences were significantly greater than those among individuals who did not have SRCD. Using the Rao–Scott Chi-Square test, SRCD was significantly correlated with CBP, gender, education, insurance, diabetes, hypertension, and obesity (*p* < 0.001 for all the pairwise comparisons). Characteristics of the study sample are presented in Table 1.

Of the participants, 21.6% experienced CBP. Among those participants with SRCD, 40.6% (95% CI: 38.6–42.6%) reported CBP while those without SRCD, 19.5% (95% CI: 18.9–20.1%) reposted CBP. Physical limitation due to chronic back pain was reported by 32.2% (95%CI: 30.1–33.8) as slight, 19.2% (95%CI: 18.0–20.4) as moderate, and 15.5% (95%CI: 14.3–16.4) as high or very high (Table 2).

The results of the unadjusted and adjusted weighted logistic regression results to investigate the association between CBP, physical limitation due to CBP, and SRCD among adult Brazilians are presented in Table 3. The weighted and adjusted odds of SRCD among adults reporting CBP were almost three times greater (weighted and adjusted odds ratio (WAOR) 2.68; 95% CI: 2.44–2.94) among Brazilian adults reporting CBP than among those who did not report CBP. Among adults with CBP, after adjusting for possible covariates, the odds of SRCD among Brazilian adults reporting slight physical limitation due to CBP were 86% greater than the odds of SRCD among those who reported no physical limitation due to CBP (WAOR 1.86; 95% CI: 1.49–2.33), while those who reported moderate physical limitation due to CBP had 149% greater odds of SRCD than among those without physical limitation due to CBP (WAOR 2.49; 95% CI: 1.98–3.14). However, when one considers those Brazilians who reported high physical limitation due to CBP, the odds of SRCD were 447% greater than the odds of SRCD among those who reported no physical limitation due to CBP (WAOR 5.47; 95% CI: 4.38–6.83).

## 4. Discussion

The goals of this study were to evaluate the association between self-reported CBP and depression and to look at a possible link between physical limitations due to CBP and depression in a very large, representative sample of Brazilian adults, on a scale that has never previously been done.

The current study found a significant association between self-reported CBP and SRCD, with those who self-reported CBP being nearly three times more likely to report SRCD when compared to those not suffering from CBP. This is in line with the review done by Sullivan et al., in 1992 of literature examining depression in patients with CBP, who determined that the prevalence of major depression in patients with CBP is around three to four times greater than that of the general population [59]. One of the biological causes of depression is the dysregulation of several neurotransmitters, including serotonin (5-hydroxytryptamin or 5-HT) and norepinephrine (NE), which are also implicated in the pathophysiology of chronic pain [60,61,62,63]. Therefore, because they share these neurotransmitters amid other biological pathways, depression and CBP should be treated simultaneously for the best outcomes [60,64]. Another interesting connection is the link between chronic fatigue, depression, and back pain. Maes outlined how depression and chronic fatigue syndrome have common aberrations in inflammatory, oxidative, and nitrosative pathways, such as systemic inflammation and its long-term sequelae, oxidative/nitrosative-induced damage to DNA, fatty acids, and proteins; dysfunctional mitochondria; lowered antioxidant levels, autoimmune responses to products of oxidation/nitrosation, and increased translocation of gram-negative bacteria [65]. On the other hand, researchers looked at back pain among college students using data from the National College Health Assessment Survey and found that chronic fatigue was one of the factors most strongly associated with back pain [66]. Chronic pain can lead to depression which can lead to more sedentary behaviors which increase pain, leading to more depression and more pain, and the cycle continues [67]. Regardless of whether chronic pain causes depression or if depression leads to chronic pain, the fact remains that they often co-exist, given the strong association between the two [7,8,9,10,11,12,13,14]. Several international studies demonstrated significant associations between CBP and depression in a combined total of 48 different countries [7,20]. Many studies in the United States have shown depression to be significantly higher among people with chronic pain than those without [11,12,68]. Rush et al., conducted a literature review in 2000 and found that CBP appears to be associated with major depression in around 50% of cases [11]. Studies in both Spain and the Netherlands have found that CBP was significantly associated with a higher risk of depression and a significantly higher risk of developing a later mood disorder, respectively [8,13]. Notwithstanding the significant amount of research globally, public health officials in Brazil had no generalizable studies that showed a link between self-reported CBP and SRCD, as previous research had been on a small scale and contradictory [24,25]. Using the results of this current study, they will be able to demonstrate the significant association between self-reported CBP and SRCD in order to guide policy change.

For the second goal of this study, the level of physical limitation due to self-reported CBP was analyzed to determine if there was a significant association with SRCD. Of the 15,317 Brazilian participants with CBP, 32.9% reported no physical limitations due to CBP, 32.4% reported low levels, 19.2% reported moderate levels, and 15.5% reported high levels. Among individuals who have SRCD and suffer from CBP, 30.9% reported high physical limitation due to CBP, a significantly greater percentage than among their counterparts (11.8%) who did not disclose SRCD. Analysis showed that Brazilians with self-reported CBP and any degree of physical limitation were nearly two to over five times more likely to have SRCD when compared to those without physical limitation. Several studies in the United States and Sweden have shown that the amount that chronic pain interfered with one’s daily activities was associated with the severity of their depression, much more so than the intensity of their pain [69,70,71]. Elfving et al., determined that ratings of activity limitation in Swedish patients with CBP were so important (and so often excluded from clinician’s evaluation of pain) that it ought to be included with ratings of pain intensity in order to obtain the clearest picture of what is going on with the patient [72]. The relationship appears to go both ways, as Bair et al., found that patients in the United States with chronic musculoskeletal pain, depression, and anxiety, experience a strong association with more severe pain and greater levels of interferences in daily activities due to pain [60].

The findings of this study need to be considered through the lens of several limitations. First, the current results come from a cross-sectional analysis. These types of studies have the inherent inability to determine causality conclusions, given that potential risk factors and outcomes were measured simultaneously through the survey. Second, due to the self-reporting nature of the responses to the 2019 PNS survey, answers may have been influenced by reporting bias, such as social desirability and recall. Third, questions regarding the effectiveness of antidepressants or other psychotropic medications were not included in the 2019 PNS, preventing the authors from adjusting for their use in this analysis.

Notwithstanding these limitations, the current study also has several strengths. First, this is the first study to investigate the association between CBP and SRCD on a national level in Brazil. Second, the final study size was very large, representing the entire Brazilian adult population, not merely a subgroup thereof, enabling the results to be generalized across the country. Third, the data was gathered via a rigorous process of sampling, collection, and validation, and the survey interviews were conducted by trained professionals at the residence of the Brazilian participants [26].

Global estimates have calculated the annual loss of productivity due to depression to be greater than $1 trillion [73,74,75,76,77]. Kessler et al., analyzed the WHO World Mental Health surveys in 2010 and found that Brazil had the highest prevalence of depression among developing countries worldwide, just over 10% in a 12-month period, meaning roughly 20 million people are affected nationally, or 6.67% of all the people in the world who suffer from depression [78]. Additionally, the estimated investments in mental health for low- and middle-income countries is less than 1% of the health budget, with only 20–40% of those who need it actually receiving treatment. On a positive note, in 36 countries (including Brazil), for every dollar invested in depression treatment from 2016 to 2030, an economic return of four dollars is expected [73,74,75,76]. Brazilian public health officials and healthcare professionals need to be aware of the significant association between CBP and SRCD, along with physical limitation due to CPB and SRCD in their country in order to develop policies to minimize the potential long-term effects of back pain and depression [73,75,76].

## 5. Conclusions

In conclusion, the present study definitively establishes an association between CBP and physical limitation due to CBP with SRCD in Brazil on a national level, which is important for several reasons, such as high medical costs, loss of productivity, low investments in mental health, and an economic return seen by treating depression. In the future, the effect modifying role of age group will need to be further investigated to expand on these findings by reflecting separately, in multiple publications, on these associations based on the individual characteristics of various age groups involved.

## Figures and Tables

**Table 1:** Characteristics of the final study sample. Brazilian National Health Survey, 2019.

Characteristics	Overall (*n* = 71,535)			Current Depression				*p*-Value *
				Yes (*n* = 7100)		No (*n* = 64,435)		
	Unweighted Counts	Weighted		Weighted		Weighted		
		Median	Interquartile Range	Median	Interquartile Range	Median	Interquartile Range	
Age at survey	71,535	42.34	30.55–55.91	43.28	31.16–55.73	42.23	30.47–55.93	0.411
Characteristics		Percent	95% CI for Percent	Percent	95% CI for Percent	Percent	95% CI for Percent	*p*-value **
Gender								
Female	36,262	51.19	50.54–51.84	72.02	70.12–73.92	48.79	48.09–49.49	<0.001
Male	35,273	48.81	48.16–49.46	27.98	26.08–29.88	51.21	50.51–51.91	
Race								
Brown	34,724	41.79	41.04–42.54	42.35	40.33–44.36	41.73	40.95–42.51	0.255
White	27,962	46.01	45.21–46.81	44.60	42.48–46.73	46.17	45.34–47.00	
Other	8849	12.20	11.74–12.65	13.05	11.62–14.48	12.10	11.62–12.58	
Education								
Less than high school	34,313	44.48	43.67–45.29	49.26	47.23–51.30	43.93	43.11–44.75	<0.001
Completed high school	20,675	31.77	31.11–32.42	28.08	26.22–29.94	32.19	31.48–32.90	
More than high school	16,547	23.75	22.95–24.56	22.66	20.66–24.66	23.88	23.03–24.73	
Health Insurance								
Yes	18,733	30.19	29.32–31.70	26.34	24.31–28.36	30.64	29.74–31.53	<0.001
No	52,802	69.81	68.93–70.68	73.66	71.64–75.69	69.36	68.47–70.26	
Diabetes								
Yes	3018	4.12	3.88–4.37	8.70	7.53–9.87	3.60	3.35–3.84	<0.001
No	68,517	95.88	95.63–96.12	91.30	90.13–92.47	96.41	96.16–96.65	
Hypertension								
Yes	9558	12.90	12.48–13.32	22.90	21.21–24.58	11.75	11.34–12.15	<0.001
No	61,977	87.10	86.69–87.52	77.10	75.42–78.79	88.25	87.85–88.66	
Physical activity for leisure								
Yes	22,120	31.90	31.22–32.58	21.96	20.09–23.83	33.05	32.34–33.76	<0.001
No	49,415	68.10	67.42–68.78	78.04	76.17–79.91	66.95	66.24–67.66	
Obesity								
Yes	15,248	21.81	21.00–22.63	27.97	25.89–30.05	21.10	20.32–21.89	<0.001
No	56,287	78.19	77.37–79.00	72.03	69.65–74.11	78.90	78.11–79.68	

* Based on unadjusted weighted logistic regression; ** Based on the Rao–Scott Chi-Square Test, which is the design-adjusted equivalent of the Pearson Chi-square test.

**Table 2:** Distribution of the exposure of interest in the final study sample. Brazilian National Health Survey, 2019.

Characteristics	Overall (*n* = 71,535)			Current Depression				*p*-Value *
				Yes (*n* = 7100)		No (*n* = 64,435)		
	Unweighted Counts	Weighted		Weighted		Weighted		
		Percent	95% CI for Percent	Percent	95% CI for Percent	Percent	95% CI for Percent	
Chronic Back Pain								
Yes	15,317	21.64	21.05–22.23	40.64	38.64–42.64	19.45	18.85–20.05	<0.001
No	56,218	78.36	77.77–78.95	59.36	57.36–61.36	80.55	79.95–81.15	
Physical limitation due to chronic back pain								
Characteristics	Overall (*n* = 15,437)			Current Depression				*p*-value **
				Yes (*n* = 2855)		No (*n* = 12,582)		
	Unweighted counts	Weighted		Weighted		Weighted		
		Percent	95% CI for Percent	Percent	95% CI for Percent	Percent	95% CI for Percent	
Physical Limitation (*n* = 15,437)								
None	4730	32.88	31.41–34.36	17.47	14.90–20.03	36.60	34.92–38.27	<0.001
Slight	5043	32.42	30.06–33.78	29.32	26.38–32.26	33.17	31.69–34.64	
Moderate	3094	19.20	18.03–20.38	22.32	19.78–24.86	18.45	17.12–19.79	
High or very high	2450	15.49	14.34–16.64	30.89	27.84–33.94	11.78	10.74–12.83	

* Based on unadjusted weighted logistic regression; ** Based on the Rao–Scott Chi-Square Test, which is the design-adjusted equivalent of the Pearson Chi-square test.

**Table 3:** The association between chronic back pain, physical limitation due to chronic back pain, and current depression among adult Brazilians: weighted univariable and multivariable results. Brazilian National Health Survey, 2019.

Exposure of Interest	Self-Reported Current Depression	
	WUOR (95% CI)	WAOR (95% CI)
Chronic back pain (*n* = 71,535)		
No	REF	REF
Yes	2.84 (2.59, 3.10)	2.68 (2.44, 2.94)
Physical limitation due to back pain (*n* = 15,317)		
None	REF	REF
Slight	1.85 (1.49, 2.31)	1.86 (1.49, 2.33)
Moderate	2.53 (2.02, 3.18)	2.49 (1.98, 3.14)
High or Very High	5.49 (4.43, 6.81)	5.47 (4.38, 6.83)

Adjustment was made for age, sex, race, education, insurance, obesity status, diabetes, hypertension, and physical activity for leisure. Bolded are significant results at the 0.05 level. WUOR = Weighted Unadjusted Odds Ratio; WAOR = Weighted Adjusted Odds Ratio; CI = Confidence Interval; REF = Reference Category.

## Data Availability

PNS data are available online for public access and use (https://www.ibge.gov.br/estatisticas/sociais/saude/9160-pesquisa-nacional-de-saude.html?=&t=microdados) (accessed on 1 March 2021).

## References

[1] World Health Organization Depression. https://www.who.int/news-room/fact-sheets/detail/depression.

[2] American Psychiatric Association (2013). Diagnostic and Statistical Manual of Mental Disorders.

[3] Nezu A.M., Ronan G.F., Meadows E.A., McClure K.S. (2002). . Practitioner’s Guide to Empirically Based Measures of Depression.

[4] Hedden S.L., Kennet J., Lipari R., Medley G., Tice P. (2015). Behavioral Health Trends in the United States: Results from the 2014 National Survey on Drug Use and Health. US Dep. Heal. Hum. Serv..

[5] Weissman M.M. (1996). Cross-National Epidemiology of Major Depression and Bipolar Disorder. JAMA J. Am. Med. Assoc..

[6] Eisendrath S.J., Cole S.A., Christensen J.F., Gutnick D., Cole M.R., Feldman M.D., Feldman M.D., Christensen J.F., Satterfield J.M. (2014). Depression. Behavioral Medicine: A Guide for Clinical Practice, 4e.

[7] Koyanagi A., Oh H., Stubbs B., Haro J.M., DeVylder J.E. (2017). Epidemiology of Depression with Psychotic Experiences and Its Association with Chronic Physical Conditions in 47 Low- and Middle-Income Countries. Psychol. Med..

[8] Fernandez M., Colodro-Conde L., Hartvigsen J., Ferreira M.L., Refshauge K.M., Pinheiro M.B., Ordoñana J.R., Ferreira P.H. (2017). Chronic Low Back Pain and the Risk of Depression or Anxiety Symptoms: Insights from a Longitudinal Twin Study. Spine J..

[9] Sagheer M.A., Khan M.F., Sharif S. (2013). Association between Chronic Low Back Pain, Anxiety and Depression in Patients at a Tertiary Care Centre. J. Pak. Med. Assoc..

[10] Banks S.M., Kerns R.D. (1996). Explaining High Rates of Depression in Chronic Pain: A Diathesis-Stress Framework. Psychol. Bull..

[11] Rush A.J., Polatin P., Gatchel R.J. (2000). Depression and Chronic Low Back Pain. Spine.

[12] Dworkin R.H., Gitlin M.J. (1991). Clinical Aspects of Depression in Chronic Pain Patients. Clin. J. Pain.

[13] van’t Land H., Verdurmen J., Ten M., Van S., De R. (2011). The Association between Chronic Back Pain and Psychiatric Disorders: Results from a Longitudinal Population-Based Study. Anxiety and Related Disorders.

[14] Gerrits M.M.J.G., van Oppen P., van Marwijk H.W.J., Penninx B.W.J.H., van der Horst H.E. (2014). Pain and the Onset of Depressive and Anxiety Disorders. Pain.

[15] Vos T., Barber R.M., Bell B., Bertozzi-Villa A., Biryukov S., Bolliger I., Charlson F., Davis A., Degenhardt L., Dicker D. (2015). Global, Regional, and National Incidence, Prevalence, and Years Lived with Disability for 301 Acute and Chronic Diseases and Injuries in 188 Countries, 1990–2013: A Systematic Analysis for the Global Burden of Disease Study 2013. Lancet.

[16] Treede R.-D., Rief W., Barke A., Aziz Q., Bennett M.I., Benoliel R., Cohen M., Evers S., Finnerup N.B., First M.B. (2015). A Classification of Chronic Pain for ICD-11. Pain.

[17] Patrick N., Emanski E., Knaub M.A. (2014). Acute and Chronic Low Back Pain. Med. Clin. N. Am..

[18] Hoy D., Bain C., Williams G., March L., Brooks P., Blyth F., Woolf A., Vos T., Buchbinder R. (2012). A Systematic Review of the Global Prevalence of Low Back Pain. Arthritis Rheum..

[19] Malta D.C., Stopa S.R., Szwarcwald C.L., Gomes N.L., Silva Júnior J.B., Reis A.A.C. (2015). dos A Vigilância e o Monitoramento Das Principais Doenças Crônicas Não Transmissíveis No Brasil—Pesquisa Nacional de Saúde, 2013. Rev. Bras. Epidemiol..

[20] Vancampfort D., Stubbs B., Koyanagi A. (2017). Physical Chronic Conditions, Multimorbidity and Sedentary Behavior amongst Middle-Aged and Older Adults in Six Low- and Middle-Income Countries. Int. J. Behav. Nutr. Phys. Act..

[21] Adilay U., Guclu B., Goksel M., Keskil S. (2018). The Correlation of SCL-90-R Anxiety, Depression, Somatization Subscale Scores with Chronic Low Back Pain. Turk. Neurosurg..

[22] Marshall P.W.M., Schabrun S., Knox M.F. (2017). Physical Activity and the Mediating Effect of Fear, Depression, Anxiety, and Catastrophizing on Pain Related Disability in People with Chronic Low Back Pain. PLoS ONE.

[23] Tsuji T., Matsudaira K., Sato H., Vietri J. (2016). The Impact of Depression among Chronic Low Back Pain Patients in Japan. BMC Musculoskelet. Disord..

[24] Trocoli T.O., Botelho R.V. (2016). Prevalência de Ansiedade, Depressão e Cinesiofobia Em Pacientes Com Lombalgia e Sua Associação Com Os Sintomas Da Lombalgia. Rev. Bras. Reumatol..

[25] Rodrigues-De-Souza D.P., Fernández-De-Las-Peñas C., Martín-Vallejo F.J., Blanco-Blanco J.F., Moro-Gutiérrez L., Alburquerque-Sendín F. (2016). Differences in Pain Perception, Health-Related Quality of Life, Disability, Mood, and Sleep between Brazilian and Spanish People with Chronic Non-Specific Low Back Pain. Braz. J. Phys. Ther..

[26] Stopa S.R., Szwarcwald C.L., de sOliveira M.M., Gouvea E.d.C.D.P., Vieira M.L.F.P., de Freitas M.P.S., Sardinha L.M.V., Macário E.M. (2020). Pesquisa Nacional de Saúde 2019: Histórico, Métodos e Perspectivas. Epidemiol. E Serviços Saúde.

[27] Szwarcwald C.L., Malta D.C., Pereira C.A., Vieira M.L.F.P., Conde W.L., de Souza Júnior P.R.B., Damacena G.N., Azevedo L.O., Azevedo e Silva G., Theme Filha M.M. (2014). Pesquisa Nacional de Saúde No Brasil: Concepção e Metodologia de Aplicação. Cien. Saude Colet..

[28] Amiel Castro R.T., Pinard Anderman C., Glover V., O’Connor T.G., Ehlert U., Kammerer M. (2017). Associated Symptoms of Depression: Patterns of Change during Pregnancy. Arch. Womens. Ment. Health.

[29] Silva M.M.d.J., Leite E.P.R.C., Nogueira D.A., Clapis M.J. (2016). Depression in Pregnancy: Prevalence and Associated Factors. Investig. Educ. Enfermería.

[30] Pearlstein T. (2015). Depression during Pregnancy. Best Pract. Res. Clin. Obstet. Gynaecol..

[31] Anderson J.E., Michalak E.E., Lam R.W. (2022). Depression in Primary Care: Tools for Screening, Diagnosis, and Measuring Response to Treatment. B. C. Med. J..

[32] Santos I.S., Tavares B.F., Munhoz T.N., de Almeida L.S.P., da Silva N.T.B., Tams B.D., Patella A.M., Matijasevich A. (2013). Sensibilidade e Especificidade Do Patient Health Questionnaire-9 (PHQ-9) Entre Adultos Da População Geral. Cad. Saude Publica.

[33] Razykov I., Ziegelstein R.C., Whooley M.A., Thombs B.D. (2012). The PHQ-9 versus the PHQ-8—Is Item 9 Useful for Assessing Suicide Risk in Coronary Artery Disease Patients? Data from the Heart and Soul Study. J. Psychosom. Res..

[34] Shin C., Lee S.-H., Han K.-M., Yoon H.-K., Han C. (2019). Comparison of the Usefulness of the PHQ-8 and PHQ-9 for Screening for Major Depressive Disorder: Analysis of Psychiatric Outpatient Data. Psychiatry Investig..

[35] Kroenke K., Spitzer R.L., Williams J.B.W., Löwe B. (2010). The Patient Health Questionnaire Somatic, Anxiety, and Depressive Symptom Scales: A Systematic Review. Gen. Hosp. Psychiatry.

[36] Kroenke K., Strine T.W., Spitzer R.L., Williams J.B.W., Berry J.T., Mokdad A.H. (2009). The PHQ-8 as a Measure of Current Depression in the General Population. J. Affect. Disord..

[37] Instituto Brasileiro de Geografia e Estatística (IBGE) Diretoria de Pesquisas Coordenação de Trabalho e Rendimento Questionário Dos Moradores Do Domicílio. https://www.pns.icict.fiocruz.br/wp-content/uploads/2021/02/Questionario-PNS-2013.pdf.

[38] Gullich I., Duro S.M.S., Cesar J.A. (2016). Depressão Entre Idosos: Um Estudo de Base Populacional No Sul Do Brasil. Rev. Bras. Epidemiol..

[39] Engin S., Ozturk M., Engin N., Baral Kulaksizoglu I. (2010). Dark Side of the Town: Depressive Symptoms in Disadvantaged Senior Citizens. J. Nutr. Health Aging.

[40] Silva M.T., Galvao T.F., Martins S.S., Pereira M.G. (2014). Prevalence of Depression Morbidity among Brazilian Adults: A Systematic Review and Meta-Analysis. Rev. Bras. Psiquiatr..

[41] Stopa S.R., Malta D.C., de Oliveira M.M., Lopes C.d.S., Menezes P.R., Kinoshita R.T. (2015). Prevalência Do Autorrelato de Depressão No Brasil: Resultados Da Pesquisa Nacional de Saúde, 2013. Rev. Bras. Epidemiol..

[42] Moreira V. (2007). Critical Phenomenology of Depression in Brazil, Chile and the United States. Lat. Am. J. Fundam. Psychopathol. Line.

[43] de Oliveira G.D., Oancea S.C., Nucci L.B., Vogeltanz-Holm N. (2018). The Association between Physical Activity and Depression among Individuals Residing in Brazil. Soc. Psychiatry Psychiatr. Epidemiol..

[44] Schuch F.B., Vancampfort D., Richards J., Rosenbaum S., Ward P.B., Stubbs B. (2016). Exercise as a Treatment for Depression: A Meta-Analysis Adjusting for Publication Bias. J. Psychiatr. Res..

[45] Schuch F.B., Vancampfort D., Rosenbaum S., Richards J., Ward P.B., Veronese N., Solmi M., Cadore E.L., Stubbs B. (2016). Exercise for Depression in Older Adults: A Meta-Analysis of Randomized Controlled Trials Adjusting for Publication Bias. Rev. Bras. Psiquiatr..

[46] Fleck M.P.d.A., Lima A.F.B.d.S., Louzada S., Schestasky G., Henriques A., Borges V.R., Camey S. (2002). Associação Entre Sintomas Depressivos e Funcionamento Social Em Cuidados Primários à Saúde. Rev. Saude Publica.

[47] Furtado R.N.V., Ribeiro L.H., de Arruda Abdo B., Descio F.J., Martucci Junior C.E., Serruya D.C. (2014). Dor Lombar Inespecífica Em Adultos Jovens: Fatores de Risco Associados. Rev. Bras. Reumatol..

[48] Jacobs J.M., Hammerman-Rozenberg R., Cohen A., Stessman J. (2006). Chronic Back Pain among the Elderly: Prevalence, Associations, and Predictors. Spine.

[49] Carey T.S., Freburger J.K., Holmes G.M., Jackman A., Knauer S., Wallace A., Darter J. (2010). Race, Care Seeking, and Utilization for Chronic Back and Neck Pain: Population Perspectives. J. Pain.

[50] Angst F., Angst J., Ajdacic-Gross V., Aeschlimann A., Rössler W. (2017). Epidemiology of Back Pain in Young and Middle-Aged Adults: A Longitudinal Population Cohort Survey from Age 27–50 Years. Psychosomatics.

[51] Zanuto E.A.C., Codogno J.S., Christófaro D.G.D., Vanderlei L.C.M., Cardoso J.R., Fernandes R.A. (2015). Prevalence of Low Back Pain and Associated Factors in Adults from a Middle-Size Brazilian City. Cien. Saude Colet..

[52] Rinaldo L., McCutcheon B.A., Gilder H., Kerezoudis P., Murphy M., Maloney P., Hassoon A., Bydon M. (2017). Diabetes and Back Pain: Markers of Diabetes Disease Progression Are Associated with Chronic Back Pain. Clin. Diabetes.

[53] Dionne C.E. (2001). Formal Education and Back Pain: A Review. J. Epidemiol. Community Health.

[54] Trask C., Bath B., McCrosky J., Lawson J. (2014). A Profile of Farmers and Other Employed Canadians with Chronic Back Pain: A Population-Based Analysis of the 2009-2010 Canadian Community Health Surveys. J. Rural Health.

[55] Rundell S.D., Sherman K.J., Heagerty P.J., Mock C.N., Dettori N.J., Comstock B.A., Avins A.L., Nedeljkovic S.S., Nerenz D.R., Jarvik J.G. (2016). Predictors of Persistent Disability and Back Pain in Older Adults with a New Episode of Care for Back Pain. Pain Med..

[56] Hardt J., Jacobsen C., Goldberg J., Nickel R., Buchwald D. (2008). Prevalence of Chronic Pain in a Representative Sample in the United States. Pain Med..

[57] Latza U., Kohlmann T., Deck R., Raspe H. (2000). Influence of Occupational Factors on the Relation between Socioeconomic Status and Self-Reported Back Pain in a Population-Based Sample of German Adults with Back Pain. Spine.

[58] (1998). Clinical Guidelines on the Identification, Evaluation, and Treatment of Overweight and Obesity in Adults—The Evidence Report. National Institutes of Health. Obes. Res..

[59] Sullivan M.J.L., Reesor K., Mikail S., Fisher R. (1992). The Treatment of Depression in Chronic Low Back Pain: Review and Recommendations. Pain.

[60] Bair M.J., Wu J., Damush T.M., Sutherland J.M., Kroenke K. (2008). Association of Depression and Anxiety Alone and in Combination with Chronic Musculoskeletal Pain in Primary Care Patients. Psychosom. Med..

[61] Moultry A.M., Poon I.O. (2009). The Use of Antidepressants for Chronic Pain. US Pharmacyst.

[62] Blier P., Abbott F.V. (2001). Putative Mechanisms of Action of Antidepressant Drugs in Affective and Anxiety Disorders and Pain. J. Psychiatry Neurosci..

[63] Jann M.W., Slade J.H. (2007). Antidepressant Agents for the Treatment of Chronic Pain and Depression. Pharmacotherapy.

[64] Thase M.E. (2016). Managing Medical Comorbidities in Patients with Depression to Improve Prognosis. J. Clin. Psychiatry.

[65] Maes M. (2011). An Intriguing and Hitherto Unexplained Co-Occurrence: Depression and Chronic Fatigue Syndrome Are Manifestations of Shared Inflammatory, Oxidative and Nitrosative (IO&NS) Pathways. Prog. Neuro-Psychopharmacol. Biol. Psychiatry.

[66] Gilkey D.P., Keefe T.J., Peel J.L., Kassab O.M., Kennedy C.A. (2010). Risk Factors Associated with Back Pain: A Cross-Sectional Study of 963 College Students. J. Manip. Physiol. Ther..

[67] Teychenne M., Ball K., Salmon J. (2010). Sedentary Behavior and Depression Among Adults: A Review. Int. J. Behav. Med..

[68] Magni G., Caldieron C., Rigatti-Luchini S., Merskey H. (1990). Chronic Musculoskeletal Pain and Depressive Symptoms in the General Population. An Analysis of the 1st National Health and Nutrition Examination Survey Data. Pain.

[69] Rudy T.E., Kerns R.D., Turk D.C. (1988). Chronic Pain and Depression: Toward a Cognitive-Behavioral Mediation Model. Pain.

[70] Von Korff M., Simon G. (1996). The Relationship between Pain and Depression. Br. J. Psychiatry.

[71] Waxman R., Tennant A., Helliwell P. (1998). Community Survey of Factors Associated with Consultation for Low Back Pain. BMJ.

[72] Elfving B., Lund I., Boström C. (2016). Ratings of Pain and Activity Limitation on the Visual Analogue Scale and Global Impression of Change in Multimodal Rehabilitation of Back Pain—Analyses at Group and Individual Level. Disabil. Rehabil..

[73] Razzouk D. (2016). Por Que o Brasil Deveria Priorizar o Tratamento Da Depressão Na Alocação Dos Recursos Da Saúde?. Epidemiol. e Serviços Saúde.

[74] Barrientos A., Møller V., Saboia J., Lloyd-Sherlock P., Mase J. (2013). ‘Growing’ Social Protection in Developing Countries: Lessons from Brazil and South Africa. Dev. South. Afr..

[75] Chisholm D., Sweeny K., Sheehan P., Rasmussen B., Smit F., Cuijpers P., Saxena S. (2016). Scaling-up Treatment of Depression and Anxiety: A Global Return on Investment Analysis. Lancet Psychiatry.

[76] World Health Organization (2015). Mental Health Atlas 2014.

[77] Dunlop D.D., Song J., Lyons J.S., Manheim L.M., Chang R.W. (2003). Racial/Ethnic Differences in Rates of Depression among Preretirement Adults. Am. J. Public Health.

[78] Kessler R.C., Birnbaum H.G., Shahly V., Bromet E., Hwang I., McLaughlin K.A., Sampson N., Andrade L.H., de Girolamo G., Demyttenaere K. (2010). Age Differences in the Prevalence and Co-Morbidity of DSM-IV Major Depressive Episodes: Results from the WHO World Mental Health Survey Initiative. Depress. Anxiety.

